# Phase I and pharmacokinetic study of the novel chemoprotector BNP7787 in combination with cisplatin and attempt to eliminate the hydration schedule

**DOI:** 10.1038/sj.bjc.6602553

**Published:** 2005-04-19

**Authors:** E Boven, M Westerman, C J van Groeningen, M Verschraagen, R Ruijter, I Zegers, W J F van der Vijgh, G Giaccone

**Affiliations:** 1 Department of Medical Oncology, VU University Medical Center, De Boelelaan 1117, 1081 HV Amsterdam, The Netherlands

**Keywords:** BNP7787, mesna, cisplatin, phase I, pharmacokinetics, saline hydration

## Abstract

BNP7787 (disodium 2,2′-dithio-bis-ethane sulphonate; Tavocept™) is a novel agent developed to protect against cisplatin (*cis*-diammine-dichloroplatinum(II))-associated chronic toxicities. In this study, we determined the recommended dose of BNP7787 when preceding a fixed dose of cisplatin, the pharmacokinetics (PKs) and the possible reduction of saline hydration. Patients with advanced solid tumours received BNP7787 in escalating doses of 4.1–41 g m^−2^ as a 15-min intravenous (i.v.) infusion followed by cisplatin 75 mg m^−2^ as a 60-min i.v. infusion together with pre- and postcisplatin saline hydration in a volume of 2200 ml; cycles were repeated every 3 weeks. PK was carried out using BNP7787, cisplatin and the combination. Twenty-five patients were enrolled in stage I of the study to determine the recommended dose of BNP7787. No dose-limiting toxicity was reached. The highest dose level of 41 g m^−2^ resulted in a low incidence of grade 2 toxicities, being nausea and vomiting, dry mouth or bad taste and i.v. injection site discomfort. Doses of BNP7787 ⩾18.4 g m^−2^ did not show a drug interaction between BNP7787 and cisplatin. In stage II of the study, patients received a fixed dose of BNP7787 of 18.4 g m^−2^ preceding cisplatin and were entered in prespecified reduced saline hydration steps. A total of 21 patients in cohorts of six to nine patients received reduced saline hydration of 1600 ml (step A), 1000 ml (step B) and 500 ml (step C). In step C, two out of six evaluable patients experienced grade 1 nephrotoxicity. Cisplatin acute toxicities in all 46 patients were as expected. Only five patients complained of paresthesias grade 1 and six developed slight audiometric changes. Partial tumour response was observed in four patients and stable disease in 15 patients. In conclusion, BNP7787 was tolerated well up to doses of 41 g m^−2^. The recommended dose of 18.4 g m^−2^ enabled safe reduction of the saline hydration schedule for cisplatin to 1000 ml. Further studies will assess whether BNP7787 offers protection against platinum-related late side effects.

The administration of cisplatin (*cis*-diammine-dichloroplatinum (II)), a well known and widely used broad-spectrum cytotoxic agent, is limited by the occurrence of serious toxicities such as nephrotoxicity. Several chemoprotective agents have been investigated in the attempt to ameliorate these side effects and potentially reduce the need for adequate hydration to prevent nephrotoxicity.

The utility and safety of an ideal chemoprotective agent include several considerations: (i) the compound should prevent, delay or mitigate drug-induced toxicities; (ii) the compound should not add important toxicities of its own; and (iii) the compound should not interfere with the antitumour activity. The mechanism of action of sulphur-containing nucleophiles that have been tested to prevent cisplatin-induced nephrotoxicity is based on inactivation of highly reactive monohydrated platinum species at the site of organ damage, that is, the kidney. Several modulating agents to prevent nephrotoxicity, such as thiosulphate (Leeuwenkamp *et al*, 1991), diethyldithiocarbamate (Gandara *et al*, 1995; Paredes *et al*, 1988), glutathione (Smyth *et al*, 1997), amifostine (Kemp *et al*, 1996; Hartmann *et al*, 2000) and mesna (Leeuwenkamp *et al*, 1991; Hausheer *et al*, 1998; Boven *et al*, 2002), have been investigated. Strong chemical reactivity of thiosulphate, diethyldithiocarbamate and mesna with cisplatin preclude their clinical usefulness (Verschraagen *et al*, 2003b). In phase III clinical trials, glutathione and amifostine have shown not to interfere with the antitumour activity of cisplatin (Kemp *et al*, 1996; Smyth *et al*, 1997; Hartmann *et al*, 2000). The only compound registered for preventing nephrotoxicity in patients with advanced cancer is amifostine (Schuchter *et al*, 2002). The present data do not support its routine use for prevention of cisplatin-associated neurotoxicity or ototoxicity (Schuchter *et al*, 2002). The administration of amifostine requires close patient monitoring as the compound can induce symptomatic hypotension in addition to emesis.

BNP7787 (disodium 2,2′-dithio-bis-ethane sulphonate; Tavocept™) is undergoing development as a novel chemoprotector against common and serious cisplatin- and paclitaxel-induced toxicities (Hausheer *et al*, 1998; Hausheer *et al*, 2003). In preclinical studies in rats and dogs, BNP7787 has been shown to protect against cisplatin-induced nephrotoxicity as confirmed by histopathology of kidney tissue. Previously, it has been reported by Ormstad and Uehara (1982) and Ormstad *et al* (1983) that BNP7787 is selectively taken up by the kidneys where it is converted into mesna. We have recently analysed the pharmacokinetics (PKs) of BNP7787 given at a dose of 1000 mg kg^−1^ intravenously (i.v.) in WARD colon tumour-bearing rats (Verschraagen *et al*, 2004a). High AUC values of BNP7787 and mesna were observed specifically in the kidney in contrast to at least 5.5-fold lower AUC values in the liver and tumour. In addition, we have shown that several detoxification routes present in the kidney, such as the endogenous thiols glutathione and cysteine, the glutaredoxin system as well as the thioredoxin system, were able to reduce BNP7787 to mesna (Verschraagen *et al*, 2004b). These findings may explain the substantial reductive capacity of the kidney, because of which mesna generated from BNP7787 can locally inactivate toxic monohydrated platinum species.

BNP7787 is the disulphide form of mesna and, therefore, does not contain a free thiol group that would interfere with the antitumour effects of cisplatin. Indeed, in preclinical studies in rats bearing WARD colon tumour as well as in nude mice bearing OVCAR-3 human ovarian cancer xenografts, it has been shown that BNP7787 did not reduce the tumour growth inhibition obtained with cisplatin (Hausheer *et al*, 1998; Boven *et al*, 2002). Moreover, BNP7787 protected against cisplatin-induced side effects in rats and dogs such as vomiting, myelosuppression and dose-limiting nephrotoxicity (Hausheer *et al*, 1998). Considerably high doses of BNP7787 could be administered i.v. without the introduction of side effects or lethal toxicity in all of these studies.

Based on the promising preclinical data of BNP7787, a phase I clinical study was performed in patients with solid tumours treated with a fixed dose of cisplatin every 3 weeks preceded by BNP7787 in escalating doses. In stage I of the study, PKs were carried out analysing cisplatin and BNP7787 alone and in combination. After having established the recommended dose of BNP7787, possible reduction of the saline hydration schedule of cisplatin was assessed in stage II of the study.

## PATIENTS AND METHODS

### Eligibility criteria

For study entry, patients were required to have a histologically or cytologically documented solid malignancy refractory to standard treatment, could not receive standard treatment or standard treatment was not available and were not previously treated with cisplatin or carboplatin. Eligibility criteria were as commonly used and included, among others, a serum creatinine ⩽120 *μ*mol l^−1^ and a calculated 24-h creatinine clearance of ⩾60 ml min^−1^. Creatinine clearance was calculated from a 24-h urine collection, but the Cockcroft–Gault formula was used in cases where the 24-h urine collection was incomplete. All patients provided written informed consent according to institutional ethical guidelines. The study was approved by the scientific and ethical review committee of the institution.

### Trial design

BNP7787 was supplied in glass vials containing 1.0, 2.0, 5.0 or 10 g of compound presented as a sterile, white powder. The agent was reconstituted by adding sterile water for injection to obtain a final concentration of 100–250 mg ml^−1^. In patients where reconstituted BNP7787 caused local irritation at the site of infusion due to low pH (pH 6.7), NaHCO_3_ 8.4% was added in a volume of 0.1–0.2 ml g^−1^ BNP7787 (dose levels 4.1–18.4 g m^−2^) to subsequent cycles. NaHCO_3_ 8.4% was increased to 0.4 ml g^−1^ BNP7787 and was admixed with the formulation routinely at higher doses of BNP7787. During the study, BNP7787 formulation was changed to a lyophilised presentation that did not cause local transient discomfort. Therefore, in stage II of the study, the addition of NaHCO_3_ 8.4% to the BNP7787 solution was omitted. After calculation of the volume of dissolved BNP7787 plus or minus NaHCO_3_, sterile NaCl 0.9% was added to a total volume of 300 ml. Cisplatin (platosin 1.0 mg ml^−1^; Pharmachemie BV, Haarlem, The Netherlands) was added to sterile NaCl 0.9% to a total volume of 180 ml.

The infusion schedule of BNP7787 and/or cisplatin for stage I of the study is shown in Table 1. Patients were treated with cisplatin 75 mg m^−2^ i.v. as a standard dose every 3 weeks. All patients received BNP7787 alone 1 week before the coadministration with cisplatin using the same administration schedule as described in Table 1. The prespecified BNP7787 starting dose was 4.1 g m^−2^. This starting dose was based on the analysis and calculation of a BNP7787: cisplatin 50 : 1 molar ratio that was required for partial cisplatin nephroprotection in rats (Hausheer *et al*, 1998). This dose represented 17% of the maximum tested dose in rats and 10.2% of that tested in Beagle dogs; these maximum tested doses were neither lethal nor toxic. Dose escalation steps of BNP7787 in cohorts of three patients each according to the protocol were 8.2, 12.3, 18.4, 27.6 and 41.0 g m^−2^. The dose of 41.0 g m^−2^ was considered to be the end point dose, since the molar ratio of BNP7787 : cisplatin of 503 : 1 would be far in excess to that required for protection in rats. Reduced dose escalation steps were to be taken in case of grade 2 BNP7787 toxicity, excluding suboptimally treated nausea and vomiting. Dose-limiting toxicity felt to be directly due to the study drug during stage I was defined as grade 4 neutropenia and any other manifestation of grade 3–4 toxicity, except for suboptimally treated nausea and vomiting. In case of dose-limiting toxicity in one patient at any dose level, a maximum of three additional patients would be treated. The maximum-tolerated dose was defined as the highest dose at which level only one out of six patients was allowed dose-limiting toxicity due to the study drug. In the event that a dose of 41.0 g m^−2^ would be reached, a total of six patients were to be studied.

Systematic reduction of saline hydration for a 75 mg m^−2^ dose of cisplatin preceded by a fixed dose of 18.4 g m^−2^ of BNP7787 was studied in steps A–D in stage II of the study. The infusion schedules are listed in Table 2. The number of patients per reduced hydration step was six. Only one out of six patients was allowed with grade 1 nephrotoxicity (creatinine upper normal value of the institution was 110 *μ*mol l^−1^) or creatinine clearance <60 ml min^−1^ on the day of the next cycle. If more than one out of six patients experienced grade 1 nephrotoxicity, the previous hydration step was considered the safe reduced hydration regimen.

Standard antiemetic prophylaxis was given to all patients receiving cisplatin and consisted of ondansetron 8 mg i.v. and dexamethasone 8 mg i.v. 30 min prior to cisplatin. In case of persistent nausea and vomiting following treatment, the antiemetic regimen was repeated after 12 h. When diuresis was less than 1000 ml over a total period of 4 h of the infusion schedule in stage I, patients received mannitol 20% and furosemide 10 mg i.v. (Table 1). In stage II of the study, no specific attention was paid to diuresis control. In case of excessive vomiting, the patients would continue i.v. infusion of NaCl 0.9% 500 ml every 6 h regardless of the stage of the protocol.

### Toxicity and response

Before the start of treatment, a medical history was taken and patients underwent a complete physical examination, including assessment of height, weight, blood pressure, temperature and performance status. The pretreatment evaluation also included a full blood cell count, a biochemical screening profile, coagulation tests, urinalysis, calculated creatinine clearance, audiogram and tumour measurements. All toxicities were assessed in accordance with the NCl Common Toxicity Criteria (original version). During the study, weekly full blood counts and serum creatinine measurements were performed. Before each treatment cycle, an interval history, a physical examination, the biochemical screening profile, coagulation tests, a creatinine clearance assessment, urinalysis and tumour measurements were performed. In case a CT scan was required for assessment of tumour response, this was carried out every three cycles. A baseline audiogram was required and a repeat audiogram was performed every three cycles.

Response to treatment was assessed using adequate imaging techniques to visualise tumour lesions. The ECOG criteria for tumour response in measurable disease were employed. Patients with an objective response or stable disease continued treatment until excessive toxicity or patient's refusal.

### Pharmacokinetics

One patient of each cohort and all patients of the cohort receiving the highest dose of BNP7787 in stage I of the study were subjected to PK analysis of plasma BNP7787, mesna, and total platinum, intact platinum, monohydrated cisplatin and unbound platinum. An extensive analysis of all PK data is presented in a separate report (Verschraagen *et al*, 2003a). A summary is given below.

In patients undergoing PK analysis, cisplatin was given alone, which was followed by the administration of BNP7787 alone 2 weeks later. A week after BNP7787 alone, the patient received the BNP7787 plus cisplatin combination. The infusion schedule, also of BNP7787 alone, was given according to Table 1 and drugs were delivered using syringe pumps.

For the high-performance liquid chromatography (HPLC) analysis of BNP7787 and its metabolite mesna, plasma samples were collected just before treatment and at 8, 15, 25, 35, 45 min and 1, 1.25, 1.75, 2.25, 4.25 and 6.25 h after the start of the BNP7787 infusion. Samples were deproteinised and the total amount of mesna and BNP7787 was measured by HPLC (with electrochemical detection) according to the method described (James *et al*, 1987; El-Yazigi *et al*, 1997; Verschraagen *et al*, 2001). For the analysis of total platinum, intact cisplatin, monohydrated cisplatin and unbound platinum, plasma samples were collected just before treatment and at 0.5, 1, 1.5, 2, 3, 4 and 6 h after the start of the cisplatin infusion. Total platinum in plasma and unbound platinum in deproteinised plasma were measured by flameless atomic absorption spectrometry (FAAS) (Verschraagen *et al*, 2003a). Intact cisplatin and monohydrated cisplatin fractions in deproteinised plasma were separated by HPLC, and subsequently, the platinum contents were measured by FAAS (Verschraagen *et al*, 2002).

Pharmacokinetic parameters, that is, final half-life (*t*_1/2_), area under the concentration–time curve over time interval 0−*t* (AUC^0−*t*^), area under the curve to infinity (AUC^*∞*^), mean residence time (MRT), total body clearance (Cl) and steady-state volume of distribution (*V*_ss_) were determined by noncompartmental analysis using the validated PK data analysis program WinNonLin Standard Edition version 1.5 (Pharsight Corporation, Mountain View, USA). The maximum plasma concentration (*C*_max_) and time of observed maximum concentration (*t*_max_) were obtained from the concentration–time data. To compare the Cl and *V*_ss_ values between patients, the values were normalised for body surface area (1.73 m^2^) and for body weight (kg), respectively.

## RESULTS

### Demographics

Twenty-five patients were enrolled in stage I of this phase I study. Patient characteristics are described in Table 3. One patient with lung cancer and a previous response to carboplatin and paclitaxel was allowed to enter the trial. All patients received at least two cycles containing cisplatin. A concern that potential mesna levels would be unacceptably high and lead to toxicity at doses of BNP7787 >18.4 g m^−2^ resulted in inclusion of two new cohorts: three patients received BNP7787 23.0 g m^−2^ and one patient received BNP7787 34.5 g m^−2^. At the BNP7787 41.0 g m^−2^ dose level, a total of six patients were treated.

A total of 21 patients were enrolled in stage II of the study. Patient characteristics are listed in Table 3. Reduced saline hydration steps A and B included six patients each. Nine patients entered step C. Three patients in step C were replaced as these were considered not to be evaluable for nephrotoxicity for the following reasons: one patient had rapidly progressive disease after cycle 1, one patient had a baseline creatinine value (109 *μ*mol l^−1^) that was considerable higher than that measured after cycle 3 (72 *μ*mol l^−1^) and one patient had variable creatinine values due to periods of poor fluid intake.

### Side effects from BNP7787

Initially, BNP7787 was supplied as a sterile powder. Most patients received treatment via a peripheral vein and occasionally complained about discomfort at the site of BNP7787 infusion (Table 4). The local i.v. site discomfort subsided promptly after completion of the BNP7787 infusion or by extending the infusion time from 15 to 30 min. BNP7787 did not cause thrombophlebitis. The local discomfort was believed to be due to the low pH (pH 6.7) of BNP7787 powder dissolved in water and diluted in NaCl 0.9%. The addition of NaHCO_3_ 8.4% alleviated this symptom promptly as well as switching to a lyophilisation process for the formulation of BNP7787 during the study. At higher doses of BNP7787 to which NaHCO_3_ was added, occasionally a patient complained about transient discomfort at the site of infusion. At the highest dose of BNP7787 of 41.0 g m^−2^, regardless of the addition of NaHCO_3_, in eight of 26 cycles local discomfort grade 1 and in two cycles grade 2 (one patient) was reported.

Side effects other than transient local discomfort from BNP7787 occurred occasionally at doses of 18.4–34.5 g m^−2^ and were rather mild (Table 4). Also, when present, complaints were reported in only one cycle and disappeared immediately after the end of the infusion. Most frequent toxicities consisted of hot flushes or a warm feeling, dry mouth or bad taste, dizziness and headache. One patient experienced nausea grade 2 and abdominal pain grade 2 in cycle 1, but had no complaints in cycle 2. At the highest dose of BNP7787 of 41.0 g m^−2^ administered to six patients, side effects were more prominent. The incidence and grade of nausea and vomiting increased as well, but did not exceed grade 2. Thus, the maximum-tolerated dose of BNP7787 was not established in this study. The BNP7787 18.4 g m^−2^ dose was recommended for further clinical studies, since (i) this dose was tolerated well, (ii) a BNP7787 : cisplatin molar ratio of 225 : 1 was considered to achieve sufficient chemoprotection, since a 50 : 1 molar ratio already partially protected nephrotoxicity in rats, and (iii) there was no evidence of a drug interaction (see below).

Based on the use of the lyophilised formulation in the course of the study, it was determined that BNP7787 18.4 g m^−2^ could be given without NaHCO_3_ in stage II of the study. Only in one cycle of a patient who received four cycles local i.v. site discomfort grade 1 necessitated prolongation of the BNP7787 infusion up to 30 min (Table 4). All other cycles were not complicated by local irritation. In stage II of the study, other side effects from BNP7787 were absent, except for one cycle in which transient hot flushes were reported.

### Side effects from cisplatin

A total of 45 patients in stages I and II of the study could be evaluated for cisplatin-induced side effects. Of these patients, three received one cycle only; the median number of cycles administered was 3 (range 1–9). Most common acute toxicities recorded were nausea, vomiting and fatigue (Table 5). Five patients suffered from grade 3 nausea, two patients from grade 3 vomiting and five patients from grade 3 fatigue. Other toxicities grade 2 encountered were a change in taste (one patient), anorexia (four patients) and weight loss (two patients). Myelosuppression, not exceeding grade 2, more often occurred in patients treated with ⩾4 cycles of cisplatin.

Out of 16 patients treated with ⩾4 cycles of cisplatin (⩾300 mg m^−2^ cumulative cisplatin dose), five complained of paresthesias grade 1. At the lowest dose level of BNP7787 4.1 g m^−2^, there were two patients with paresthesias: in one patient this started at cycle 5, but symptoms did not worsen within the 9-cycle period of treatment, and the other patient had neurological complaints at the 8th and last cycle. One patient on BNP7787 12.3 g m^−2^ dose level had paresthesias at the 6th and last cycle. At the BNP7787 23 g m^−2^ dose level, one patient had complaints at cycle 6 out of a total of seven cycles. A last patient at the 41.0 g m^−2^ BNP7787 dose level had paresthesias recorded in the 9th and last cycle. Another five patients who received ⩾6 cycles of cisplatin had no subjective peripheral neurotoxicity.

All 16 patients treated with ⩾4 cycles of cisplatin had a repeat audiogram and five out of eight patients treated with ⩾6 cycles had a second repeat audiogram. In six patients, slight hearing loss was recorded after cycle 3 (one patient), cycle 4 (two patients), cycle 5 (two patients) and after cycle 6 (one patient). Four of these patients had occasional tinnitus from cycle 2 (one patient), cycle 4 (two patients) and cycle 7 (one patient) onwards. Three other patients complained of occasional tinnitus after one cycle (one patient) and after two cycles (two patients) of cisplatin without changes on the audiogram, but were not treated further.

In stage I of the study, a total of 11 patients received ⩾4 cycles and eight were treated with ⩾6 cycles cisplatin preceded by BNP7787. Only one patient at the BNP7787 4.1 g m^−2^ level showed an increase in creatinine grade 1 at cycle 6. Nephrotoxicity in this patient did not deteriorate further during the 9-cycle treatment period.

### Reduction of the hydration schedule

In stage II of the study, four decremental steps were prespecified to evaluate the nephroprotective effect of BNP7787 18.4 g m^−2^ prior to cisplatin using a reduced volume of saline hydration that is otherwise required for cisplatin administration. None of the patients continued additional 500 ml NaCl 0.9% every 6 h for excessive vomiting.

**Figure 1:**
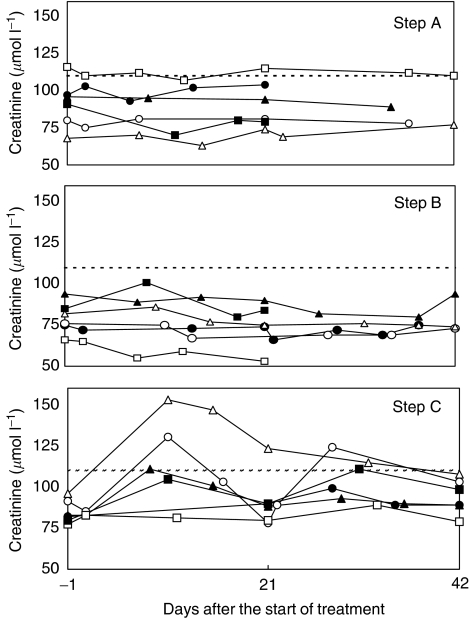
Creatinine values of patients treated with cisplatin 75 mg m^−2^ preceded by BNP7787 18.4 g m^−2^ every 3 weeks and saline hydration schedules according to steps A, B and C (Table 2). Day 21 and day 42 represent the start of cycle 2 and cycle 3, respectively. Dotted line: upper normal value of the institution. Each symbol represents one patient.

Figure 1 step A depicts the creatinine values measured in the first two cycles of cisplatin given with hydration according to step A. Two out of six patients received only one cycle due to rapidly progressive disease. The maximum number of cycles administered to patients in cohort step A was 3. No patient experienced nephrotoxicity grade 1 in cohort A. In cohort B, there was no evidence of cisplatin nephrotoxicity on the day starting cycle 2 or 3 (Figure 1 step B). One patient, however, had a baseline creatinine value of 85 *μ*mol l^−1^, which showed a transient increase to 101 *μ*mol l^−1^. Owing to rapidly progressive disease, this patient discontinued treatment. Two patients who received four and six cycles, respectively, according to hydration step B did not show evidence of cisplatin-induced renal toxicity.

Cohort C consisted of nine patients because of replacement of one patient who had rapidly progressive disease and two patients who were not evaluable for cisplatin-induced nephrotoxicity (see demographics). All six evaluable patients received at least two cycles, of which four showed a transient increase in creatinine up to grade 1 in between cycles. Two patients experienced nephrotoxicity grade 1 at the end of the first (creatinine 123 *μ*mol l^−1^) and the second cycle (creatinine clearance 58.5 ml min^−1^), respectively (Figure 1 step C). One of these patients was further hydrated according to step B from cycles 2–5; baseline creatinine of 96 *μ*mol l^−1^ increased to 128 *μ*mol l^−1^ by the end of cycle 5, but recovered to 97–105 *μ*mol l^−1^ in the week thereafter. The other patient was hydrated according to step B from cycle 3 and according to stage I of the study from cycles 4–8; baseline creatinine of 91 *μ*mol l^−1^ increased to 126 *μ*mol l^−1^ by the end of cycle 8 and recovered to 109 *μ*mol l^−1^ within 3 weeks. One other patient received six cycles in which the transient increase in creatinine in between cycles reached grade 1 at the start of cycle 6. Thus, in cohort step C, two out of six patients reached the stopping rule of step-wise reduction in saline hydration in the first two treatment cycles. Therefore, step D was cancelled.

### Tumour response

Most patients who entered the study had an advanced solid malignancy for which cisplatin is not a standard treatment, except three patients with lung cancer and six patients with head-and-neck cancer (Table 3). A partial tumour response was noted in four patients, one patient with melanoma, one with head-and-neck cancer, one with two adenocarcinoma primaries originating from lung and breast and one with a bile duct cancer. Stable disease was noted in 15 patients of the following tumour types: colorectal cancer (three patients), adenocarcinoma of unknown primary (three patients), head-and-neck cancer (four patients), and lung cancer, chondrosarcoma, mesothelioma, bile duct cancer and renal cell cancer (one patient each).

### Pharmacokinetics

**Figure 2:**
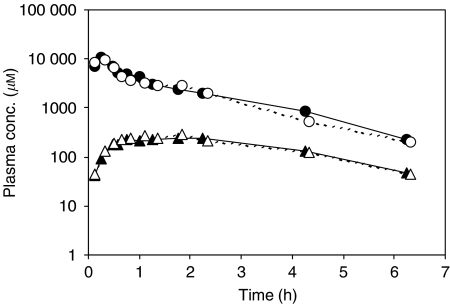
Semilogarithmic plasma concentration–time plots of BNP7787 (•, ○) and mesna (▴, Δ) of the patient who received a 15-min infusion of 18.4 g m^−2^ BNP7787 alone (closed symbols and solid line) and in combination with 1-h i.v. infusion of 75 mg m^−2^ cisplatin (open symbols and dotted line).

Detailed analysis of PKs of BNP7787 and cisplatin has been reported elsewhere (Verschraagen *et al*, 2003a) and can be summarised as follows. Linear PKs was observed for both the AUC^*∞*^ and *C*_max_ of BNP7787 and mesna enabling normalisation of values for the dose. The mean normalised AUC^*∞*^ of mesna was approximately 8% of the normalised AUC^*∞*^ of BNP7787. The mean *t*_1/2_ of BNP7787 in plasma was 1.4 h, while that of mesna was 2.7 h. MRT of mesna was approximately two-fold longer than that of BNP7787 (ie 4.5 and 1.9 h, respectively). The *V*_ss_ and Cl of BNP7787 were approximately 0.26 l kg^−1^ and 9.2 l h^−1^ 1.73 m^−2^, respectively. The BNP7787 and mesna curves after the BNP7787 administration were similar to the curves obtained after BNP7787 followed by cisplatin. Semilogarithmic plasma concentration–time plots for BNP7787 and mesna observed in the patient who received 18.4 g m^−2^ BNP7787 alone and in combination with 75 mg m^−2^ cisplatin are shown in Figure 2.

**Figure 3:**
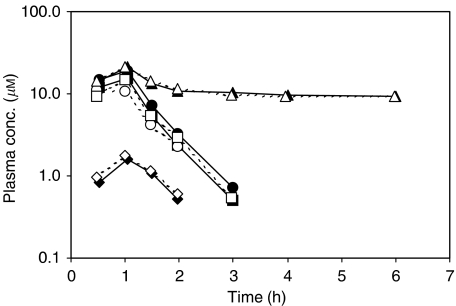
Semilogarithmic plasma AUCs of total platinum (▴, Δ), unbound platinum (•, ○) intact cisplatin (▪, □) and monohydrated cisplatin (⧫, ◊) of the patient who received a 1-h i.v. infusion of 75 mg m^−2^ cisplatin alone (closed symbols and solid line) and in combination with a 15-min i.v. infusion of 18.4 g m^−2^ BNP7787 (open symbols and dotted line).

The concentration–time curves of total platinum, unbound platinum, monohydrated cisplatin and intact cisplatin were similar after cisplatin administration or preceded by BNP7787. Only in patients sampled at a dose of 4.1, 8.2 and 12.3 g m^−2^, BNP7787 administration showed a slight tendency to lower the plasma concentrations of intact cisplatin. BNP7787 did not influence the intact cisplatin concentrations at doses of ⩾18.4 g m^−2^. Semilogarithmic plasma concentration–time plots for total platinum, unbound platinum, monohydrated cisplatin and intact cisplatin observed in the patient who received 75 mg m^−2^ cisplatin alone and in combination with 18.4 g m^−2^ BNP7787 are shown in Figure 3.

## DISCUSSION

We performed a phase I study to explore the safety, the maximum-tolerated dose and the recommended dose of i.v. BNP7787 when preceding a fixed dose of cisplatin. Side effects attributable to the study drug were minor and included occasional discomfort at the site of the infusion and a low incidence of nausea and vomiting, dry mouth or bad taste, not exceeding grade 2. BNP7787-related toxicities disappeared promptly after the end of the infusion. Dose-limiting toxicity was not reached, since a dose of 41.0 g m^−2^ of BNP7787 was considered as the end point of the study. Pharmacokinetics at doses of BNP7787 ⩾18.4 g m^−2^ did not show the presence of a drug interaction between BNP7787 and cisplatin (Verschraagen *et al*, 2003a).

BNP7787 has been developed specifically as a protector against late side effects from cisplatin such as nephrotoxicity. In stage I of the study, a total of 11 patients received ⩾4 cycles and eight were treated with ⩾6 cycles cisplatin 75 mg m^−2^ i.v. preceded by BNP7787. Only one patient at the lowest BNP7787 level of 4.1 g m^−2^ experienced nephrotoxicity grade 1 that did not progress within the period of nine treatment cycles given (675 mg m^−2^ cisplatin total dose). From literature data, it is expected that the incidence and the severity of nephrotoxicity rapidly increases at a cumulative dose of cisplatin of ⩾450 mg m^−2^. As an example, the incidence of nephrotoxicity grades 1–3 was 82% in ovarian cancer patients treated with six to 12 cycles of cisplatin 75 mg m^−2^ plus cyclophosphamide 750 mg m^−2^ every 3 weeks (Neijt *et al*, 1987). The same schedule given for a maximum of six cycles in a similar patient population was reported to induce grade 1–4 nephrotoxicity in only 7% of patients (McGuire *et al*, 1996). Cisplatin 80 mg m^−2^ day 2 plus gemcitabine 1200 mg m^−2^ days 1 and 8 given every 3 weeks up to six cycles to patients with advanced non-small-cell lung cancer induced renal side effects grades 1–4 in 6.1% of patients (Mazzanti *et al*, 2003). Although the number of patients treated with cisplatin at a cumulative dose ⩾450 mg m^−2^ according to stage I of our study was small, the absence of nephrotoxicity when given concurrently with BNP7787 ⩾18.4 g m^−2^, being a molar ratio of 225 : 1, may suggest some degree of nephroprotection.

At the time of initial introduction of cisplatin for clinical use, the optimal single dose was 15–20 mg m^−2^ given every 3 weeks without hydration (Prestayko *et al*, 1979). The incidence of nephrotoxicity was 32% in subsequent phase II studies. Since then, the incidence and severity of renal toxicity has been substantially reduced by pre- and posthydration saline infusions up to a volume of 4000 ml and addition of mannitol and furosemide. The disadvantage of hyperhydration for patients is the overnight stay in the clinic as the duration of the infusion may be as long as 24 h and the additional costs for monitoring and care. Furthermore, patients with chronic heart failure or unstable cardiac condition may not receive hyperhydration. In stage II of our study in which cisplatin 75 mg m^−2^ was preceded by BNP7787 18.4 g m^−2^, the hydration schedule could be reduced to a total volume of 1000 ml given over a period of less than 3 h (step B).

Five of 16 patients on ⩾4 treatment cycles in stages I and II of the study experienced paresthesias grade 1. The incidence and severity of cisplatin-induced neuropathy largely depends on the cumulative dose, the onset of symptoms being seen at a total dose of cisplatin 225–450 mg m^−2^ (Cavaletti *et al*, 1997). In the ovarian cancer patients treated with cisplatin 75 mg m^−2^ plus cyclophosphamide 750 mg m^−2^ every 3 weeks for six to 12 cycles, the incidence of peripheral neuropathy grades 1–3 was 46% (Neijt *et al*, 1987), and when given for only six cycles, this was grades 1–4 in 21% of patients (McGuire *et al*, 1996). In non-small-cell lung cancer patients treated with cisplatin 80 mg m^−2^ day 2 plus gemcitabine 1200 mg m^−2^ days 1 and 8 every 3 weeks up to six cycles, the incidence of peripheral neuropathy grades 1–4 was 12.9% (Mazzanti *et al*, 2003). The cumulative dose of cisplatin at the start of paresthesias in our study varied between 375 and 675 mg m^−2^, and notably none exceeded grade 1. It should be emphasised that three out of five patients with neurological complaints were treated at the lower dose levels of BNP7787 4.1–12.3 g m^−2^. Thus, the low incidence and low grade of paresthesias induced by cisplatin in patients on ⩾4 cycles of cisplatin in our study suggests that BNP7787 has a role in the protection against cisplatin-induced neurotoxicity. Of interest, in two other phase I trials, BNP7787 administration substantially prevented and mitigated the severity of paclitaxel-induced neurotoxicity (Hausheer *et al*, 2003) as well as cisplatin-induced neurotoxicity (FH Hausheer, unpublished data).

Long-term use of cisplatin can cause ototoxicity. Tinnitus may occur as early as after the first cycle (Cavaletti *et al*, 1997). After the third 3-weekly cycle of cisplatin 75 mg m^−2^ plus cyclophosphamide 750 mg m^−2^ in the study by Cavaletti *et al* (1997), hearing loss was detected in the 8000-Hz frequency range on the audiogram in 13 out of 19 ovarian cancer patients. Deterioration of hearing loss was evident in 15 out of their 17 patients examined after six cycles. All 16 patients in our study treated with ⩾4 cycles of cisplatin had a repeat audiogram. In six patients, slight hearing loss was recorded, and four of these patients complained of occasional tinnitis. Since five out of eight patients treated with ⩾6 cycles had a second repeat audiogram, no definitive conclusion can be drawn on the long-term prevention of this side effect.

In conclusion, BNP7787 preceding cisplatin was tolerated very well. Since a low number of patients treated with cisplatin ⩾300 mg m^−2^ suffered from nephrotoxicity and neurotoxicity not exceeding grade 1, BNP7787 might protect against these cisplatin-induced toxicities. BNP7787 at a dose of 18.4 g m^−2^ may enable cisplatin infusion with drastically reduced saline hydration (1000 ml of fluid) in the outpatient clinic. BNP7787 does not appear to interfere with the antitumour activity of cisplatin, since several objective responses were observed in this phase I trial. Currently, phase III trials are underway to determine the usefulness of BNP7787 in the prevention of paclitaxel- and cisplatin-associated neurotoxicity without reduction of the antitumour effects.

## Figures and Tables

**Table 1:** Infusion schedule of BNP7787 and/or cisplatin in stage I of the study

**Time period (min)**	**Volume (ml)**	**Contents of the infusion fluid**
90	1000	NaCl 0.9%, 20 mmol KCl, 2 g MgSO_4_
10	100	Mannitol 20%
15	300	BNP7787 (by syringe pump if PK)
60	180	Cisplatin 75 mg/m^2^ (by syringe pump if PK)
90	1000	NaCl 0.9%, 20 mmol KCl, 2 g MgSO_4_
10	100	Mannitol 20% (+furosemide 10 mg)^a^

BNP7787=disodium 2,2′-dithio-bis-ethane sulphonate; cisplatin=*cis*-diammine-dichloroplatinum(II); PK=pharmacokinetics.

a At the end of the infusion over a total period of 4 h diuresis should be at least 1000 ml; if not, furosemide 10 mg i.v. should be administered.

**Table 2:** Infusion schedule of BNP7787 and cisplatin in stage II of the study

**Time period (min)**	**Volume (ml)**	**Contents of the infusion fluid**
*Step A*		
90	1000	NaCl 0.9%, 20 mmol KCl, 2 g MgSO_4_
10	100	Mannitol 20%
15	300	BNP7787 18.4 mg/m^2^
60	180	Cisplatin 75 mg/m^2^
30	500	NaCl 0.9%, 10 mmol KCl, 1 g MgSO_4_
		
*Step B*		
60	500	NaCl 0.9%
15	300	BNP7787 18.4 g/m^2^
60	180	Cisplatin 75 mg/m^2^
30	500	NaCl 0.9%
		
*Step C*		
30	500	NaCl 0.9%
15	300	BNP7787 18.4 g/m^2^
60	180	Cisplatin 75 mg/m^2^
		
*Step D*		
15	300	BNP7787 18.4 g/m^2^
60	180	Cisplatin 75 mg/m^2^

BNP7787=disodium 2,2′-dithio-bis-ethane sulphonate; cisplatin=*cis*-diammine-dichloroplatinum(II).

**Table 3:** Patient characteristics

	**Stage I**	**Stage II**
*Total no. of patients*	25	21
Male/female	14/11	14/7
		
*Age (years)*		
Median	53	58
Range	37–64	42–70
		
*ECOG performance status*		
Median	1	1
Range	0–2	0–2
		
*Prior therapy*		
Chemotherapy	14	8
Radiotherapy	3	5
Chemotherapy+radiotherapy	2	5
None	6	3
		
*Tumour type*		
Colorectal	8	7
Lung	3	
Melanoma	2	1
Mesothelioma	2	
Stomach	1	1
Bile duct	2	1
Pancreas	1	1
Head and neck	2	4
Breast		1
Kidney		1
Unknown primary	2	1
Other	2	3

ECOG=Eastern Cooperative Oncology Group.

**Table 4:** Number of cycles with grade 1 side effects from BNP7787

	**Stage I**	**Stage I**	**Stage II**
	**4.1–34.5 g/m^2^**	**41.0 g/m^2^**	**18.4 g/m^2^**
**Side effect**	**(78 cycles)**	**(26 cycles)**	**(62 cycles)**
Hot flushes or warm feeling	2	10	1
Dry mouth or bad taste	2	6 (1 grade 2)	
Headache	1	10	
Dizziness	2	11	
Hypotension	2		
Perspiration	1		
Myalgia	1		
Nausea	1 (1 grade 2)	7 (5 grade 2)	
Vomiting		8 (4 grade 2)	
Abdominal pain	1 (1 grade 2)		
Vigors		1	
Fatigue		2	
Pain chest		1	
Paresthesias		1	
Local i.v. site discomfort	9 (4 grade 2)	10 (2 grade 2)	1

BNP7787=disodium 2,2′-dithio-bis-ethane sulphonate; i.v.=intravenous.

**Table 5:** Common acute side effects from cisplatin at various dose levels of BNP7787 and hydration schedules

				**Nausea**			**Vomiting**			**Fatigue**			**Leucopenia**			**Neutropenia**			**Thrombopenia**		
**BNP7787 (mg/m^2^)**	**Hydration schedule**	**No. of patients**	**No. of cycles**	**1**	**2**	**3**	**1**	**2**	**3**	**1**	**2**	**3**	**1**	**2**	**3**	**1**	**2**	**3**	**1**	**2**	**3**
4.1	Stage I	3	26	1	1	1	1	2	0	2	0	1	1	2	0	0	2	0	0	2	0
8.2	Stage I	3	9	3	0	0	1	0	0	0	0	1	0	0	0	0	0	0	0	0	0
12.3	Stage I	3	10	3	0	0	2	1	0	0	1	0	0	0	0	0	0	0	0	0	0
18.4	Stage I	3	7	1	2	0	2	0	0	0	0	0	0	0	0	0	0	0	2	0	0
23.0	Stage I	3	15	1	2	0	1	1	0	0	2	0	0	2	0	2	0	0	0	0	0
27.6	Stage I	3	9	0	2	1	1	1	0	0	1	0	1	0	0	0	0	0	1	0	0
34.5	Stage I	1	2	1	0	0	0	0	0	0	1	0	0	0	0	0	0	0	0	0	0
41.0	Stage I	6	26	3	3	0	2	4	0	0	3	0	2	1	0	1	1	0	1	0	0
18.4	II – step A	6	12	2	3	1	2	3	0	1	1	2	1	0	0	1	0	0	2	0	0
18.4	II – step B	6	19	1	2	1	0	1	2	0	3	0	0	2	0	1	1	0	2	0	0
18.4	II – step C	8	31	1	4	1	2	5	0	2	2	1	1	0	0	0	0	0	1	0	0

BNP7787=disodium 2,2′-dithio-bis-ethane sulphonate; cisplatin=*cis*-diammine-dichloroplatinum(II).

## References

[bib1] Boven E, Verschraagen M, Hulscher TM, Erkelens CA, Hausheer FM, Pinedo HM, Van der Vijgh WJ (2002) BNP7787, a novel protector against platinum-related toxicities, does not affect the efficacy of cisplatin or carboplatin in human tumour xenografts. Eur J Cancer 38: 1148–1156 12008205 10.1016/s0959-8049(02)00036-9

[bib2] Cavaletti G, Bogliun G, Crespi V, Marzorati L, Zincone A, Marzola M, Rota S, Galli A, Tredici P, Tredici G (1997) Neurotoxicity and ototoxicity of cisplatin plus paclitaxel in comparison to cisplatin plus cyclophosphamide in patients with epithelial ovarian cancer. J Clin Oncol 15: 199–206 8996143 10.1200/JCO.1997.15.1.199

[bib3] El-Yazigi A, Ernst P, Al-Rawithi S, Legayada E, Raines DA (1997) Pharmacokinetics of mesna and dimesna after simultaneous intravenous bolus and infusion administration in patients undergoing bone marrow transplantation. J Clin Pharmacol 37: 618–624 9243355 10.1002/j.1552-4604.1997.tb04344.x

[bib4] Gandara DR, Nahhas WA, Adelson MD, Lichtman SM, Podczaski ES, Yanovich S, Homesley HD, Braly P, Ritch PS, Weisberg SR, Williams L, Diasio RB, Perez EA, Karp D, Reich SD, McCarroll K, Hoff JV (1995) Randomized placebo-controlled multicenter evaluation of diethyldithiocarbamate for chemoprotection against cisplatin-induced toxicities. J Clin Oncol 13: 490–496 7844610 10.1200/JCO.1995.13.2.490

[bib5] Hartmann JT, Fels LM, Knop S, Stolt H, Kanz L, Bokemeyer C (2000) A randomized trial comparing the nephrotoxicity of cisplatin/ifosfamide based combination chemotherapy with or without amifostine in patients with solid tumors. Invest New Drugs 18: 281–289 10958599 10.1023/a:1006490226104

[bib6] Hausheer FH, Kanter P, Cao S, Haridas K, Seetharamulu P, Reddy D, Petluru P, Zhao M, Murali D, Saxe JD, Yao S, Martinez N, Zukowski A, Rustum YM (1998) Modulation of platinum-induced toxicities and therapeutic index: mechanistic insights and first- and second-generation protecting agents. Semin Oncol 25: 584–599 9783598

[bib7] Hausheer FH, Kochat H, Parker AR, Ding D, Yao S, Hamilton SE, Petluru PN, Leverett BD, Bain SH, Saxe JD (2003) New approaches to drug discovery and development: a mechanism-based approach to pharmaceutical research and its application to BNP7787, a novel chemoprotective agent. Cancer Chemother Pharmacol 52(Suppl 1): S3–S15 12819940 10.1007/s00280-003-0653-5

[bib8] James CA, Mant TGK, Rogers HJ (1987) Pharmacokinetics of intravenous and oral sodium 2-mercaptoethane sulphonate (mesna) in normal subjects. Br J Clin Pharmacol 23: 561–568 3109461 10.1111/j.1365-2125.1987.tb03092.x PMC1386192

[bib9] Kemp G, Rose P, Lurain J, Berman M, Manetta A, Roullet B, Homesley H, Belpomme D, Glick J (1996) Amifostine pretreatment for protection against cyclophosphamide-induced and cisplatin-induced toxicities: Results of a randomized control trial in patients with advanced ovarian cancer. J Clin Oncol 14: 2101–2112 8683243 10.1200/JCO.1996.14.7.2101

[bib10] Leeuwenkamp OR, Neijt JO, Van der Vijgh WJ, Pinedo HM (1991) Reaction kinetics of cisplatin and its monoaquated species with the modulating agents (di)mesna and thiosulphate. Eur J Cancer 27: 1243–1247 1835593 10.1016/0277-5379(91)90090-z

[bib11] Mazzanti P, Massacesi C, Rocchi MBL, Mattioli R, Lippe P, Trivisonne R, Buzzi F, De Signoribus G, Tuveri G, Rossi G, Di Lullo L, Sturba F, Morale D, Catanzani S, Pilone A, Bonsignori M, Battelli T (2003) Randomized, multicenter, phase II study of gemcitabine plus cisplatin *versus* gemcitabine plus carboplatin in patients with advanced non-small cell lung cancer. Lung Cancer 41: 81–89 12826316 10.1016/s0169-5002(03)00140-5

[bib12] McGuire WP, Hoskins WJ, Brady MF, Kucera PR, Partridge EE, Look KY, Clarke-Pearson DL, Davidson M (1996) Cyclophosphamide and cisplatin compared with paclitaxel and cisplatin in patients with stage III and stage IV ovarian cancer. N Engl J Med 334: 1–6 7494563 10.1056/NEJM199601043340101

[bib13] Neijt JP, Ten Bokkel Huinink WW, van der Burg MEL, van Oosterom AT, Willemse PHB, Heintz APH, van Lent M, Trimbos JB, Bouma J, Vermorken JB, Van Houwelingen JC (1987) Randomized trial comparing two combination chemotherapy regimens (CHAP-5 v CP) in advanced ovarian carcinoma. J Clin Oncol 5: 1157–1168 3114434 10.1200/JCO.1987.5.8.1157

[bib14] Ormstad K, Orrenius S, Låstbom T, Uehara N, Pohl J, Stekar J, Brock N (1983) Pharmacokinetics and metabolism of sodium 2-mercaptoethanesulfonate in the rat. Cancer Res 43: 333–338 6401168

[bib15] Ormstad K, Uehara N (1982) Renal transport and disposition of Na-2-mercaptoethane sulfonate disulfide (dimesna) in the rat. FEBS Lett 150: 354–358 6819161 10.1016/0014-5793(82)80767-9

[bib16] Paredes J, Hong WK, Felder TB, Dimery IW, Choksi AJ, Newman RA, Castellanos AM, Robbins KT, McCarthy K, Atkinson N, Kramer AM, Hersh EM, Goepfert H et al (1988) Prospective randomized trial of high-dose cisplatin and fluorouracil infusion with or without sodium diethyldithiocarbamate in recurrent and/or metastatic squamous cell carcinoma of the head and neck. J Clin Oncol 6: 955–962 2836565 10.1200/JCO.1988.6.6.955

[bib17] Prestayko AW, D'Aoust JC, Issell BF, Crooke ST (1979) Cisplatin (*cis*-diamminedichloroplatinum II). Cancer Treat Rev 6: 17–39 378370 10.1016/s0305-7372(79)80057-2

[bib18] Schuchter LM, Hensley ML, Meropol NJ, Winer EP (2002) 2002 Update of recommendations for the use of chemotherapy and radiotherapy protectants: clinical practice guidelines of the American Society of Clinical Oncology. J Clin Oncol 20: 2895–2903 12065567 10.1200/JCO.2002.04.178

[bib19] Smyth JF, Bowman A, Perren T, Wilkinson P, Prescott RJ, Quinn KJ, Tedeschi M (1997) Glutathione reduces the toxicity and improves quality of life of women diagnosed with ovarian cancer treated with cisplatin: results of a double-blind, randomised trial. Ann Oncol 8: 569–573 9261526 10.1023/a:1008211226339

[bib20] Verschraagen M, Boven E, Ruijter R, van der Born K, Berkhof J, Hausheer FH, van der Vijgh WJF (2003a) Pharmacokinetics and preliminary clinical data of the novel chemoprotectant BNP7787 and cisplatin and their metabolites. Clin Pharm Ther 74: 765–771 10.1016/S0009-9236(03)00150-4 12891226

[bib21] Verschraagen M, Boven E, Torun E, Erkelens CA, Hausheer FH, van der Vijgh WJF (2004a) Pharmacokinetic behaviour of the chemoprotectants BNP7787 and mesna after an i.v. bolus injection in rats. Br J Cancer 90: 1654–1659 15083199 10.1038/sj.bjc.6601719 PMC2410273

[bib22] Verschraagen M, Boven E, Torun E, Hausheer FH, Bast A, Van der Vijgh WJF (2004b) Possible (enzymatic) routes and biological sites for metabolic reduction of BNP7787, a new protector against cisplatin-induced side-effects. Biochem Pharmacol 68: 493–502 15242815 10.1016/j.bcp.2004.04.005

[bib23] Verschraagen M, Kedde MA, Hausheer FH, Van der Vijgh WJF (2003b) The chemical reactivity of BNP7787 and its metabolite mesna with the cytostatic agent cisplatin: comparison with the nucleophiles thiosulfate, DDTC, glutathione and its disulfide GSSG. Cancer Chemother Pharmacol 51: 499–504 12715205 10.1007/s00280-003-0610-3

[bib24] Verschraagen M, Van der Born K, Zwiers THU, Van der Vijgh WJF (2002) Simultaneous determination of intact cisplatin and its metabolite monohydrated cisplatin in human plasma. J Chromatogr B 772: 273–281 10.1016/s1570-0232(02)00108-3 12007772

[bib25] Verschraagen M, Zwiers THU, De Koning P, Welink J, Van der Vijgh WJF (2001) Quantification of BNP7787 (dimesna) and its metabolite mesna in human plasma and urine by high performance liquid chromatography with electrochemical detection. J Chromatogr B 753: 293–302 10.1016/s0378-4347(00)00563-6 11334343

